# Cytoplasmic FOXP1 expression is correlated with ER and calpain II expression and predicts a poor outcome in breast cancer

**DOI:** 10.1186/s13000-018-0715-y

**Published:** 2018-05-30

**Authors:** Bao-Hua Yu, Bai-Zhou Li, Xiao-Yan Zhou, Da-Ren Shi, Wen-Tao Yang

**Affiliations:** 1 Department of Pathology, Fudan University Shanghai Cancer Center, Dong-an Road 270, Xuhui District, Shanghai, 200032 China; 2 Department of Oncology, Shanghai Medical College, Fudan University, Shanghai, China; 3 Department of Pathology, the Second Affiliated Hospital of Zhejiang University, 88 Jiefang Road, Hangzhou, 310009 China

**Keywords:** Breast cancer, FOXP1, ER, Calpain II, AKT pathway, Immunohistochemistry, Survival

## Abstract

**Background:**

Nuclear forkhead box protein P1 (N-FOXP1) expression in invasive breast cancer has been documented in the literature. However, the FOXP1 expression patterns at different stages of breast cancer progression are largely unknown, and the significance of cytoplasmic FOXP1 (C-FOXP1) expression in breast cancer has not been well illustrated. The aims of this study were to investigate FOXP1 expression patterns in invasive ductal carcinoma (IDC), ductal carcinoma in situ (DCIS), atypical ductal hyperplasia (ADH) and usual ductal hyperplasia (UDH), and to analyze the clinicopathological relevance of C-FOXP1 and its prognostic value in IDC.

**Methods:**

N-FOXP1 and C-FOXP1 expression in cases of IDC, DCIS, ADH and UDH was determined using immunohistochemistry. The correlation between C-FOXP1 expression and clinicopathological parameters as well as the overall survival (OS) and disease-free survival (DFS) rates of patients with IDC were analyzed.

**Results:**

Exclusive N-FOXP1 expression was found in 85.0% (17/20), 40.0% (8/20), 12.2% (5/41) and 10.8% (9/83) of UDH, ADH, DCIS, and IDC cases, respectively, and exclusive C-FOXP1 expression was observed in 0% (0/20), 0% (0/20), 4.9% (2/41), and 31.3% (26/83) of the cases, respectively. Both N- and C-FOXP1 staining were observed in 15.0% (3/20), 60.0% (12/20), 82.9% (34/41) and 48.2% (40/83) of the above cases, respectively, while complete loss of FOXP1 expression was observed in only 9.6% (8/83) of IDC cases. Estrogen receptor (ER) expression in C-FOXP1-positive IDC cases (31/66, 47.0%) was significantly lower than that in C-FOXP1-negative cases (13/17, 76.5%) (*p* = 0.030). Calpain II expression was observed in 83.3% (55/66) of C-FOXP1-positive IDC cases, which was significantly higher than that in C-FOXP1-negative cases (9/17, 52.9%) (*p* = 0.007). Calpain II was significantly associated with pAKT (*p* = 0.029), pmTOR (*p* = 0.011), p4E-BP1 (*p* < 0.001) and p-p70S6K (*p* = 0.003) expression levels. The 10-year OS and DFS rates of the C-FOXP1-positive patients were 60.5% and 48.7%, respectively, both of which were lower than those of the C-FOXP1-negative patients (93.3, 75.3%). The OS curve showed a dramatic impact of C-FOXP1 status on OS (*p* = 0.045).

**Conclusions:**

Cytoplasmic relocalization of FOXP1 protein was a frequent event in breast IDC. Calpain II might play an important role in nucleocytoplasmic trafficking of FOXP1 and the AKT pathway might be involved in this process. C-FOXP1 expression was inversely associated with ER expression and might be a predictor of poor OS in patients with IDC.

## Background

Breast cancer is the most common female malignancy and also the second leading cause of cancer-related death among women worldwide [1]. However, its molecular pathogenesis is largely unknown, and clinically useful prognostic and predictive parameters, apart from human epidermal growth factor receptor-2 (HER2), estrogen receptor (ER), progesterone receptor (PR) and lymph node status, are still insufficient, emphasizing the need for further investigating additional prognostic biomarkers and potential targets for selective therapies.

*The forkhead box protein P1* (*FOXP1*) gene, locating on 3p14.1, is a member of the forkhead/winged helix transcription factor family, and the FOXP1 protein is widely expressed in normal tissues [2–5]. FOXP1 protein subcellular localization varies between different tissues. A predominant nuclear FOXP1 (N-FOXP1) distribution has been identified in the kidney, thyroid, cerebellum, tonsil, blood, thymus, spleen, skin, pancreas and colon, whereas cytoplasmic FOXP1 (C-FOXP1) labeling was observed in other epithelial tissues, such as the stomach [3]. Altered FOXP1 expression is also associated with various types of tumors [6]. For example, N-FOXP1 protein is up-regulated in diffuse large B-cell lymphoma (DLBCL) and extranodal marginal zone or mucosa-associated lymphoid tissue (MALT) lymphoma [7], while loss of N-FOXP1 expression characterizes malignancy in certain solid tumors, including endometrial and prostate tumors as well as familial and sporadic breast cancer [3, 8–10]. The presence of N-FOXP1 expression is correlated with ERα and/or ERβ reactivity in invasive breast cancers [8, 11, 12]. A correlation between N-FOXP1 and ERα has also been observed in endometrial adenocarcinoma [9]. Loss of FOXP1 nuclear expression is the most striking observation, and cytoplasmic expression is noted more frequently in endometrial adenocarcinoma according to the literature. However, to date, data regarding C-FOXP1 expression in breast cancer are limited, and its clinicopathological relevance, including its correlation with ER expression, has not been well illustrated.

The oncogenic functions of FOXP1 in tumors, such as DLBCL, MALT lymphoma, and hepatocellular and renal cell carcinoma, have been well documented [4, 13, 14]. On the other hand, FOXP1 might attenuate tumorigenicity to exert a tumor-suppressive effect in other tumors, such as neuroblastoma and prostate cancer [4, 15–17]. Therefore, FOXP1 is associated with cancer patient prognosis in a context-dependent manner [4, 18]. Overall, FOXP1 positivity, with either nuclear or an unspecified distribution, is associated with favorable survival in patients with breast cancer [4, 8, 18]. Nevertheless, the prognostic value of C-FOXP1 expression in breast cancer patients has not been discussed in the literature.

The underlying mechanisms of the nucleocytoplasmic shuttling of FOXP1 in breast cancer are largely unknown. The calpains are a family of calcium-dependent cysteine proteases that function in a wide range of important cellular activities [19]. The ubiquitously expressed family members, μ-calpain (calpain I) and m-calpain (calpain II), are the most extensively studied calpains [20, 21]. Calpain II activity is subject to many forms of posttranslational control in vivo, including translocation from the cytosol to the membrane [22]. Calpains are implicated in the cleavage of several apoptosis-associated proteins, notably Bax, Bcl2, JNK and JUN, amongst others [19, 23], and are involved in the regulation of some cell cycle progression-associated proteins, such as p21, cyclin D1, and p27Kip1 [24, 25]. For example, calpains may cleave Bcl-2 and Bid and permit translocation of Bax and Bid to the mitochondria, amplifying the apoptotic signaling pathway in cancer cells [26, 27]. In addition, calpains can mediate p27Kip1 degradation, and nuclear export might be necessary for this process [24]. The PI3K/AKT/mTOR signaling pathway, including its downstream molecules p4E-BP1 and p-p70S6K, plays a crucial role in initiation and progression of breast tumorigenesis and drug resistance [28, 29]. Calpain II might promote breast cancer cell proliferation through the PI3K/AKT signaling pathway [30]. However, whether calpain II plays a role in FOXP1 regulation in breast cancer has not yet been documented.

Herein, we investigated both the cytoplasmic and nuclear expression of FOXP1 protein in cases of invasive ductal cancer (IDC) or ductal carcinoma in situ (DCIS), as well as in atypical ductal hyperplasia (ADH) and usual ductal hyperplasia (UDH) of the breast, and further analyzed the association of C-FOXP1 expression with ER, calpain II and other clinicopathological parameters in IDC, and also evaluated the prognostic value of C-FOXP1.

## Methods

### Patient selection and tissue microarray (TMA) construction

Altogether, 83 cases of IDC, 41 of DCIS, 20 of ADH, and 20 of UDH were retrieved from the archival files of the Department of Pathology, Fudan University Shanghai Cancer Center (Shanghai, China). The study was approved by the Institutional Review Board of Fudan University Shanghai Cancer Center (Shanghai Cancer Center Ethics Committee). H&E-stained sections for each case were independently reviewed by two of the authors (BHY and BZL) according to the criteria described in the World Health Organization classification of tumors of the breast [31].

Clinical data, including follow-up data, were available for all of the 83 IDC cases. For TMA construction, H&E-stained sections from each formalin-fixed paraffin-embedded block were first observed to define representative tumor cell-rich areas and then 2 representative 0.6 mm cores were obtained from each IDC case and inserted into a recipient paraffin block in a grid pattern using a tissue arrayer (Beecher Instruments, Silver Spring, MD, USA). Four-micrometer-thick sections were then cut from the TMA blocks for routine hematoxylin and eosin (H&E) staining and immunohistochemical procedures. The H&E-stained sections were used to verify the adequate representation of the diagnostic biopsies.

### Immunohistochemical staining

Following deparaffinization and heat-mediated antigen retrieval, immunohistochemical staining was carried out using an Envision system (DAKO, Glostrup, Denmark) with primary antibodies against FOXP1 (JC12, AbD Serotec, Oxford, UK), ER (SP1, Roche Tucson, AZ, USA), calpain II (CAPN2, Sigma, St. Louis, MO, USA), HER2 (4B5, Roche Tucson), pAKT (736E11, Cell signaling, Danvers, MA, USA), pmTOR (49F9, Cell signaling), p4E-BP1 (53H11, Cell signaling) and p-p70S6K (49D7, Cell signaling). The stained sections were then counterstained with hematoxylin. Appropriate positive and negative controls were carried out simultaneously for all stains.

The immunostaining results were reviewed by 2 independent qualified pathologists. Nuclear and cytoplasmic tumor cell staining for FOXP1 protein was analyzed separately. FOXP1 nuclear expression was scored using the following system: negative = 0; weak/focal staining = 1; strong focal/wide spread moderate staining = 2; or strong/widespread staining = 3. Tumors that scored 2 or 3 were considered positive for N-FOXP1 [8]. Scoring of C-FOXP1, calpain II, pAKT, pmTOR, p4E-BP1, p-p70S6K were performed in terms of the staining intensity (intensity score: 0, none; 1, weak; 2, moderate; and 3, strong) and the proportion of positive tumor cells (proportion score: less than 5% positive cells were scored as 0; 5 to 25% as 1; 26 to 50% as 2; 51 to 75% as 3; greater than 75% as 4) according to previously described scoring methods with a slight modification [9, 32, 33]. These two scores were then multiplied to yield the final score. A final score of ≥3 was defined as positive.

The status of ER, PR and HER2 were evaluated using the scoring criteria of the American Society of Clinical Oncology (ASCO)/College of American Pathologists (CAP) guideline [34, 35]. Staining was considered positive for ER when nuclear staining was present in more than 1% of the tumor cells. Immunohistochemistry for HER2 as 3+ was defined as positive. For cases of HER2 IHC 2+, Abbott-Vysis HER2 FISH assay was employed to further confirm the status of HER2 gene amplification.

### Statistical analysis

All statistical analyses were performed using the SPSS software package (SPSS version 19.0, SPSS Inc., Chicago, IL, USA). Categorical variables were compared with a χ^2^ test, and measurement data were analyzed using Pearson correlation analysis. Overall survival (OS) was defined as the interval from the initial diagnosis to the time of death or the last contact. Surviving patients were censored at the last known date of contact. Disease-free survival (DFS) was determined according to the time from diagnosis to the time of recurrence or the last contact. Patient survival was estimated using the Kaplan-Meier method and was compared by means of a log-rank test. All *p*-values were two sided, and a *p*-value < 0.05 was considered statistically significant.

## Results

### FOXP1 protein expression patterns in UDH, ADH, DCIS, and IDC of the breast

**Fig. 1:**
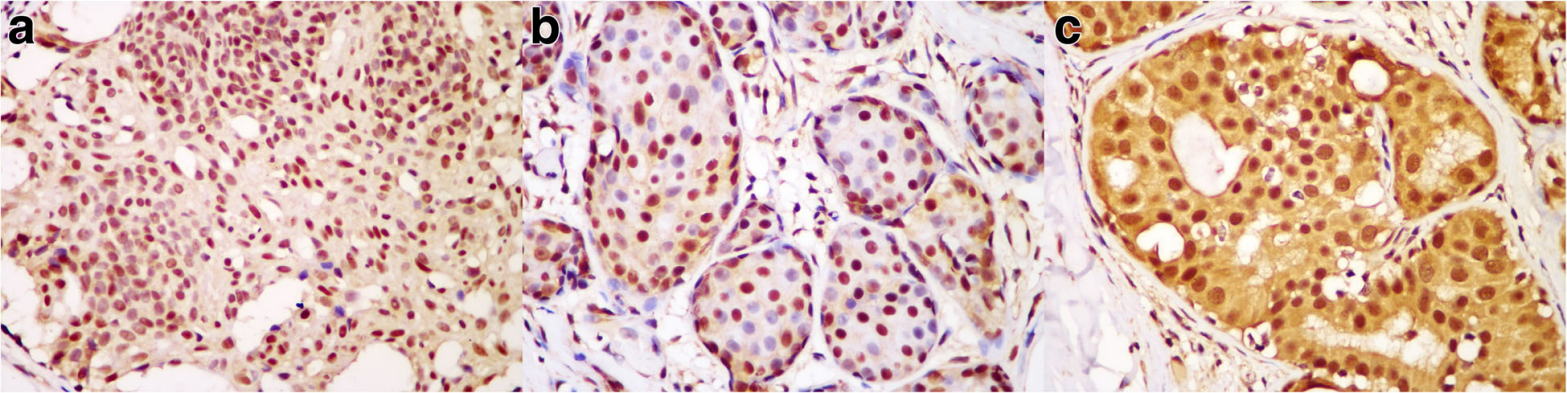
Representative cases of FOXP1 expression in UDH and ADH. FOXP1 positive staining was located in the nuclei of ductal cells in UDH (**a** ×400). In ADH, FOXP1 positivity was observed in the nuclei of tumor cells (**b** ×400) or in both the nuclei and the cytoplasm (**c** ×400)

**Fig. 2:**
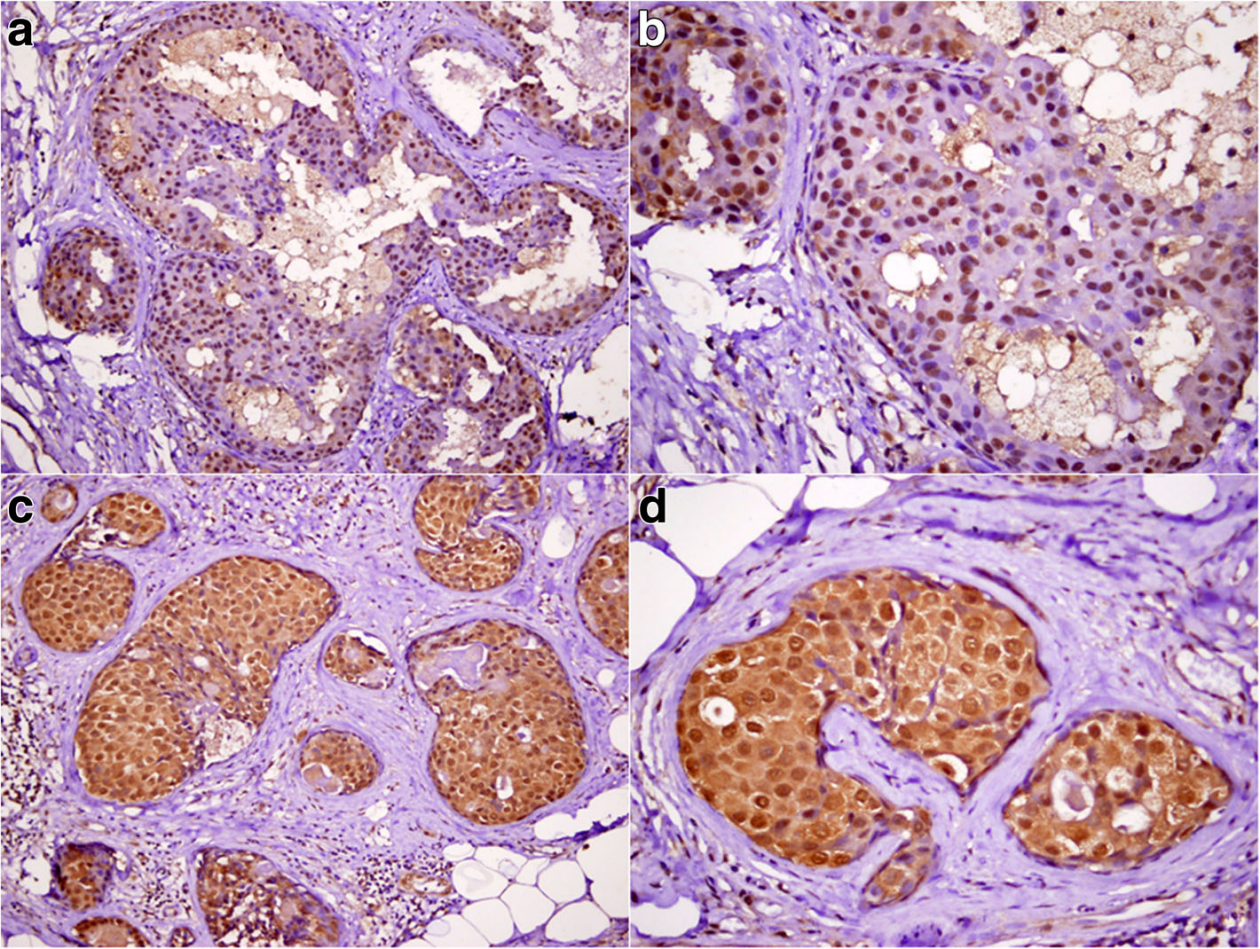
FOXP1 expression patterns in DCIS. FOXP1 immunostaining was observed in the nuclei of tumor cells (**a** ×200, **b** ×400) or both the nuclei and cytoplasm (**c** ×200, **d** ×400)

**Fig. 3:**
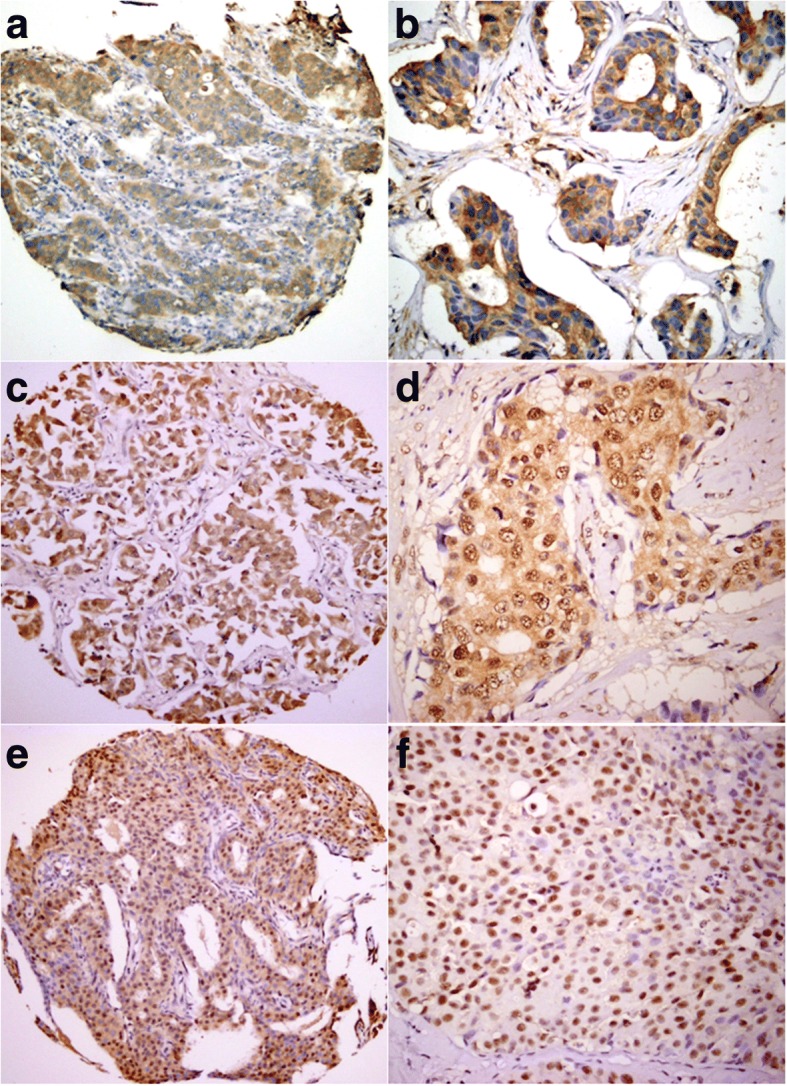
FOXP1 expression patterns in IDC. The FOXP1 protein expression patterns in IDC tumor cells ranged from exclusive cytoplasmic (**a**, TMA; **b** ×400) to mixed nuclear/cytoplasmic (**c**, TMA; **d** ×400) and to exclusive nuclear (**e**, TMA; **f** ×400)

Most UDH cases (17/20, 85%) showed uniform strong N-FOXP1 staining and the other 3 cases (15.0%) showed both N- and C-FOXP1 staining (Fig. 1). As for ADH group, 40.0% (8/20) of cases demonstrated nuclear positivity and 60.0% (12/20) showed both nuclear and cytoplasmic positivity. Nevertheless, solely cytoplasmic staining was not found in these two groups. In comparison, exclusive N-FOXP1 expression was present only in 12.2% (5/41) of DCIS cases, while both N- and C-FOXP1 expression was observed in the majority of this group (34/41, 82.9%), and the remaining 2 cases (4.9%) revealed exclusive cytoplasmic labeling (Fig. 2). The FOXP1 expression pattern in IDC samples varied. In this group, exclusive cytoplasmic staining (26/83, 31.3%) was more frequently observed than solely nuclear staining (9/83, 10.8%), both nuclear and cytoplasmic staining accounted for 48.2% (40/83) of cases and complete loss of expression was observed in 8 cases (9.6%) (Fig. 3). Moreover, exclusive cytoplasmic FOXP1 expression was more common in IDC than that in DCIS, ADH and UDH (31.3% vs 4.9, 0 and 0%). The FOXP1 expression patterns were significantly different in UDH, ADH, DCIS and IDC (*p* < 0.001, Table 1). Even within a single case, different lesions showed diverse FOXP1 staining patterns. For example, clear nuclear and cytoplasmic staining was observed in the DCIS region, while solely nuclear staining was seen in the epithelium of adjacent benign ducts.

**Table 1:** FOXP1 protein expression patterns in different breast lesions

Breast lesions	Total number *n* = 164	FOXP1 expression patterns			
		Exclusive nuclear expression n (%)	Both nuclear and cytoplasmic expression n (%)	Exclusive cytoplasmic expression n (%)	Complete loss of expression n (%)
UDH	20	17 (85.0)	3 (15.0)	0 (0)	0 (0)
ADH	20	8 (40.0)	12 (60.0)	0 (0)	0 (0)
DCIS	41	5 (12.2)	34 (82.9)	2 (4.9)	0 (0)
IDC	83	9 (10.8)	40 (48.2)	26 (31.3)	8 (9.6)

### Correlation between C-FOXP1 expression and clinicopathological variables in breast IDC

**Fig. 4:**
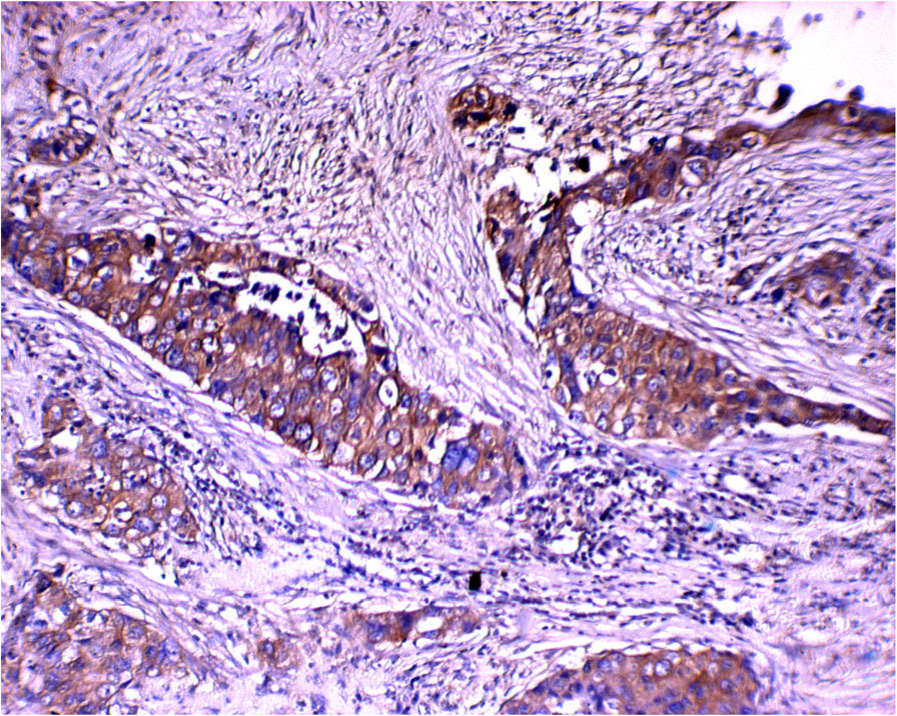
Calpain II-positive staining was found in the cytoplasm of IDC tumor cells (× 400)

The identified associations between C-FOXP1 expression and histopathological and clinical variables in IDC are shown in Table 2. ER staining was observed in 47.0% (31/66) of C-FOXP1-positive staining cases, which was lower than that in C-FOXP1-negative cases (13/17, 76.5%) (*p* = 0.030). Calpain II expression was found in 83.3% (55/66) of C-FOXP1 positive cases, compared with 52.9% (9/17) of C-FOXP1-negative ones, and the difference was statistically significant (*p* = 0.007, Fig. 4). Nevertheless, there was no significant relevance between C-FOXP1 expression and patient age, tumor size, grade, tumor stage, nodal status, distant metastasis or HER2 expression (all *p* > 0.05). pAKT, pmTOR, p4E-BP1 and p-p70S6K, as key members in the AKT pathway, was expressed in 72.3% (60/83), 74.7% (62/83), 69.9% (58/83) and 73.5% (61/83) of IDC cases in the current series, respectively. Interestingly, calpain II expression was statistically associated with the expression of pAKT (*p* = 0.029), pmTOR (*p* = 0.011), p4E-BP1 (*p* < 0.001) and p-p70S6K (*p* = 0.003, Table 3).

**Table 2:** The correlation between cytoplasmic FOXP1 expression and clinicopathological parameters in IDC cases

Clinicopathological parameters	Cytoplasmic FOXP1 expression		
	Positive (%) *n* = 66	Negative (%) *n* = 17	*P* value
ER			**0.030** ^**a**^
Positive	31 (47.0)	13 (76.5)	
Negative	35 (53.0)	4 (23.5)	
HER2			0.443
Positive	22 (33.3)	4 (23.5)	
Negative	44 (66.7)	13 (76.5)	
Calpain II			**0.007** ^**a**^
Positive	55 (83.3)	9 (52.9)	
Negative	11 (16.7)	8 (47.1)	
pAKT			0.863
Positive	48 (72.7)	12 (70.6)	
Negative	18 (27.3)	5 (29.4)	
pmTOR			0.093
Positive	52 (78.8)	10 (58.8)	
Negative	14 (21.2)	7 (41.2)	
p4E-BP1			0.607
Positive	47 (71.2)	11 (64.7)	
Negative	19 (28.8)	6 (35.3)	
p-p70S6K			0.363
Positive	50 (75.8)	11 (64.7)	
Negative	16 (24.2)	6 (35.3)	
Stage			0.562
Stage I	7 (10.6)	1 (5.9)	
Stage II	38 (57.6)	13 (76.5)	
Stage III	21 (31.8)	3 (17.6)	
Grade			0.325
Grade I	16 (24.2)	3 (17.6)	
Grade II	38 (57.6)	9 (52.9)	
Grade III	12 (18.2)	5 (29.4)	
Nodal status			0.090
Positive	49 (74.2)	9 (52.9)	
Negative	17 (25.8)	8 (47.1)	
Distant metastasis			0.230
Positive	26 (39.4)	4 (23.5)	
Negative	40 (60.6)	13 (76.5)	
Tumor size			0.907
≦4 cm	30 (45.5)	8 (47.1)	
> 4 cm	36 (54.5)	9 (52.9)	
Age			0.762
≦55 yrs	44 (66.7)	12 (70.6)	
> 55 yrs	22 (33.3)	5 (29.4)	

^a^Statistically significant *p* values are in bold

**Table 3:** The correlation between calpain II expression and clinicopathological parameters in IDC cases

Clinicopathological parameters	Calpain II expression		
	Positive (%) *n* = 64	Negative (%) *n* = 19	*P* value
ER			0.580
Positive	35 (54.7)	9 (47.4)	
Negative	29 (45.3)	10 (52.6)	
HER2			0.597
Positive	21 (32.8)	5 (26.3)	
Negative	43 (67.2)	14 (73.7)	
pAKT			**0.029** ^**a**^
Positive	50 (78.1)	10 (52.6)	
Negative	14 (21.9)	9 (47.4)	
pmTOR			**0.011** ^**a**^
Positive	52 (81.3)	10 (52.6)	
Negative	12 (18.8)	9 (47.4)	
p4E-BP1			**< 0.001** ^**a**^
Positive	52 (81.3)	6 (31.6)	
Negative	12 (18.8)	13 (68.4)	
p-p70S6K			**0.003** ^**a**^
Positive	52 (81.3)	9 (47.4)	
Negative	12 (18.8)	10 (52.6)	
Stage			0.773
Stage I	7 (10.9)	1 (5.3)	
Stage II	37(57.8)	14 (73.7)	
Stage III	20 (31.3)	4 (21.2)	
Grade			0.568
Grade I	16 (25.0)	3 (15.8)	
Grade II	35 (54.7)	12 (63.2)	
Grade III	13 (20.3)	4 (21.1)	
Nodal status			0.876
Positive	45 (70.3)	13 (68.4)	
Negative	19 (29.7)	6 (31.6)	
Distant metastasis			0.122
Positive	26 (40.6)	4 (21.1)	
Negative	38 (59.4)	15 (78.9)	
Tumor size			**0.013** ^**a**^
≦4 cm	34 (53.1)	4 (21.1)	
> 4 cm	30 (46.9)	15 (78.9)	
Age			0.653
≦55 yrs	44 (68.8)	12 (63.2)	
> 55 yrs	20 (31.3)	7 (36.8)	

^a^Statistically significant *p* values are in bold

### Correlation between C-FOXP1 expression and the survival of patients with breast IDC

**Fig. 5:**
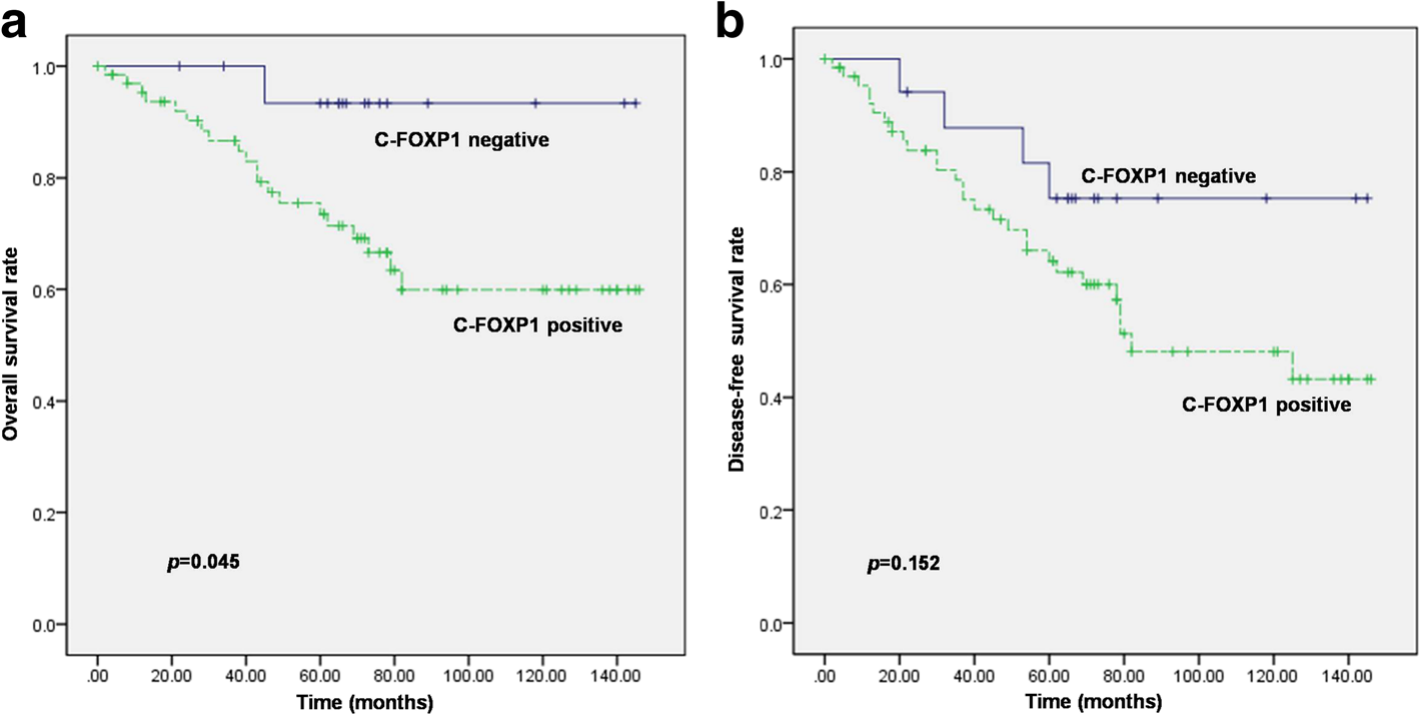
Kaplan-Meier survival curves of patients with IDC according to C-FOXP1 expression. Patients with positive C-FOXP1 immunoreactivity showed inferior OS (**a**) and DFS (**b**) compared with C-FOXP1-negative patients, although the difference in DFS was not statistically significant

Among the 83 patients with breast IDC, the follow-up period ranged from 2 to 146 months (median, 67 months), and there were 32 relapses and 21 deaths. Twenty out of 66 C-FOXP1-positive patients died of disease, and 28 had relapses, whereas 1 and 4 out of 17 C-FOXP1-negative patients died or relapsed, respectively. The 10-year OS and DFS rates of the C-FOXP1-positive patients were 60.5 and 48.7%, respectively, both of which were lower than that of the C-FOXP1-negative patients (93.3, 75.3%). The OS curve showed that C-FOXP1 status had an impact on outcome (*p* = 0.045). The DFS curve suggested that patients with C-FOXP1-negative IDCs demonstrated longer DFS than those with C-FOXP1-positive disease, but the result did not reach statistical significance (*p* = 0.152). Survival curves stratified for C-FOXP1 expression are shown in Fig. 5.

## Discussion

Although N-FOXP1 expression in breast cancer has been documented in several studies, the expression patterns of FOXP1 protein at different stages of breast cancer progression, including DCIS and IDC, and in ADH and UDH lesions, have not yet been clearly demonstrated. In the current study, heterogeneous FOXP1 expression patterns were observed in the above-mentioned cases. While FOXP1 staining was predominantly localized in the nuclei in UDH, the FOXP1 nuclear distribution gradually decreased from ADH, DCIS to IDC, and the cytoplasmic staining increased. These results were consistent with the previous reported heterogeneous expression pattern of FOXP1, in terms of the proportion of positive cells, the staining intensity, and subcellular localization [3, 36]. Our observations strongly indicated that FOXP1 expression might shift from the nucleus to the cytoplasm during breast tumorigenesis, and therefore, cytoplasmic mislocalization of FOXP1 is suggested play an important role in breast cancer progression. Similarly, subcellular localization has been suggested to play a distinct role in the pathogenesis of endometrial cancer [9]. Banham et al. also revealed that FOXP1 protein expression levels and compartmentalization varied depending on the cancer stage, although their sample sizes for each tumor were quite small [3].

Studies on the mechanisms of FOXP1 subcellular relocalization in breast cancer are very few. Calpain II has been implicated in mediating cell differentiation, necrosis, migration, and metastasis [19, 22]. Several studies, although limited, have investigated the aberrant expression and role of calpain II in breast cancer [19, 21, 25, 30, 37–40]. High calpain II expression has been established in triple-negative and basal-like IDC and calpain II might promote breast cancer cell proliferation through the AKT signaling pathway [21, 30]. Calpain-mediated cleavage of β-catenin and E-cadherin may lead to aberrant stabilization of the proteins and promote tumorigenesis in breast cancer cells [38, 39]. In addition, Ho et al. suggested that the FOXO3a subcellular location was skewed toward nuclear localization in calpain II-deficient cells [20]. To date, we are not aware of any literature establishing the relevance between calpain II and FOXP1 protein in breast cancer. An unexpected but important finding in the current study was that C-FOXP1 expression was remarkably associated with calpain II in IDC. Moreover, in IDC samples in our series, calpain II was strongly correlated with the important molecules in AKT pathway, including pAKT, mTOR, p4E-BP1 and p-p70S6K. The PI3K/AKT/p70S6K signaling pathway, which has been reported to be involved in the nucleus-cytoplasm shuttling of FOXO1, another forkhead protein family member, was previously shown to participate in FOXP1 regulation in breast cancer [30, 41]. Taken together, we speculate that calpain II might play an important role in the subcellular regulation of FOXP1, and the AKT pathway might be involved in this process. Further investigations are merited to confirm this hypothesis and thoroughly explore the underlying mechanisms.

While N-FOXP1 was positively associated with ERα as well as ERβ expression in breast cancer according to the previous studies [8, 11, 12, 30, 42], the clinicopathological relevance of C-FOXP1 positivity in IDC has not been addressed until now. For the first time, we identified an inverse correlation between C-FOXP1 expression and ER expression in IDC. Our results are in line with those of a previous report by Giatromanolaki et al. concerning endometrial carcinoma [9]. They demonstrated that loss of ERα expression was a frequent event in cases with C-FOXP1 expression or loss of FOXP1 expression in endometrial carcinoma. Given that nucleus-cytoplasm shuttling might be an important event in the carcinogenesis, the interaction between ER and C-FOXP1 expression might be more clinically significant than that originally established between ER and N-FOXP1, and its biological significance should be further explored [8, 11, 30, 43, 44].

Previous studies have demonstrated that loss of FOXP1 expression is associated with a poor prognosis in primary invasive and familial breast cancer [11]. For example, both Fox et al. and Ijichi et al. indicated that FOXP1 immunoreactivity predicted better relapse-free survival but not OS in breast cancer patients [6, 8]. Moreover, FOXP1 immunoreactivity may predict a favorable prognosis for breast cancer patients treated with tamoxifen [6, 42, 44]. However, in previous studies, the FOXP1 protein was either located in the nuclei, or its subcellular location was not specified; nonetheless, cytoplasmic FOXP1 localization might play a large role in cancer cell biology because nuclear expression is characteristic of normal breast tissues [9]. Therefore, the prognostic impact of C-FOXP1 overexpression in IDC patients might be meaningful. Our results indicate for the first time that C-FOXP1 immunoreactivity is associated with an unfavorable OS and slightly inferior DFS in patients with breast IDC. Similarly, exclusive C-FOXP1 expression in early endothelial carcinoma has been linked with deep myometrial invasion and conferred a slightly worse outcome, despite an insignificant difference [9]. Hu et al. demonstrated that increased cytoplasmic FOXP1 expression was correlated with increased tumor grade but was not significantly associated with chemotherapy resistance and prognosis [32]. Our results provide reliable evidence regarding the prognostic importance of C-FOXP1 overexpression in breast cancer, which should be further confirmed with a much larger case series.

## Conclusions

In summary, cytoplasmic relocalization of the FOXP1 protein is a frequent event in breast cancer. For the first time, we found that C-FOXP1 expression was dramatically associated with ER expression and correlated with reduced OS in patients with breast IDC. Our results indicated that C-FOXP1 might be important in both the pathogenesis and prognosis of breast cancer patients. Another noteworthy finding was that calpain II might be involved in FOXP1 trafficking from the nucleus to the cytoplasm, which might be mediated by the AKT pathway. Further investigations are needed to better understand the biological role of FOXP1 expression in breast cancer development and progression and to provide better strategies for prognosis prediction and therapeutic intervention in breast cancer.

## Abbreviations

C-FOXP1: Cytoplasmic FOXP1
DCIS: Ductal carcinoma in situ
DFS: Disease-free survival
DLBCL: Diffuse large B-cell lymphoma
ER: Estrogen receptor
FOXP1: Forkhead box protein P1
HER2: Human epidermal growth factor receptor-2
IDC: Invasive ductal carcinoma
MALT: Mucosa associated lymphoid tissue
N-FOXP1: Nuclear FOXP1
OS: Overall survival
PR: Progesterone receptor
TMA: Tissue microarray
